# The Role of Topical Povidone-Iodine in the Management of Infectious Keratitis: A Pilot Study

**DOI:** 10.3390/jcm11030848

**Published:** 2022-02-05

**Authors:** Emilio Pedrotti, Erika Bonacci, Raphael Kilian, Camilla Pagnacco, Adriano Fasolo, Marco Anastasi, Gessica Manzini, Francesca Bosello, Giorgio Marchini

**Affiliations:** 1 Ophthalmic Unit, Department of Neurosciences, Biomedicine and Movement Sciences, University of Verona, 37129 Verona, Italy; emilio.pedrotti@univr.it (E.P.); bonaccierika89@gmail.com (E.B.); camilla.pagnacco@gmail.com (C.P.); adriano.fasolo@yahoo.it (A.F.); marcoanastasi94@gmail.com (M.A.); gessica.manzini@aovr.veneto.it (G.M.); francesca.bosello87@gmail.com (F.B.); giorgio.marchini@univr.it (G.M.); 2 Research Center, The Veneto Eye Bank Foundation, 30174 Venezia, Italy

**Keywords:** infectious keratitis, corneal swab, time-to-result for pathogen identification, PVP-I, personalized therapy

## Abstract

The aim of this prospective explorative study was to evaluate the safety and the effectiveness of topical polyvinylpyrrolidone-iodine (PVP-I) administered during the time-to-results period for pathogen identification and susceptibility testing in patients with infectious keratitis (IK). A corneal swab (CS) for antimicrobial evaluation was performed at enrollment (T0) and topical 0.66%-PVP-I was administered until the laboratory results were available (T1). Ulcer and infiltrate areas and infiltrate depths were compared between T0 and T1 (i.e., time-to-result period). Patients were then shifted to a specific antimicrobial therapy and followed up until resolution of their infiltrates (Tlast-TL). Twenty-five eyes were enrolled, and none showed clinical worsening leading to protocol withdrawal. At T1, ulcer and infiltrate areas showed significant improvement in Gram-positive IK (*n* = 13–52%; *p* = 0.027 and *p* = 0.019, respectively), remained stable in fungal IK (*n* = 5–20%; both *p* = 0.98) and increased in those with Gram-negative bacteria (*n* = 4–16%; *p* = 0.58 and *p* = 0.27). Eyes with negative cultures (*n* = 3–12%) showed complete resolution at T1 and did not initiate any additional antimicrobial therapy. The administration of 0.66% PVP-I during the time-to-result period seems to be a safe strategy in patients with IK while often sparing broad-spectrum antimicrobial agents. In addition, it showed to be effective in eyes with a Gram-positive bacterial infection.

## 1. Introduction

Infectious keratitis (IK) is one of the leading causes of monocular blindness worldwide and represents a real challenge for ophthalmologists [1].

Identification of the causative organism is central to the successful medical treatment of IK and to avoid their dreaded sequalae [2]. Establishing the etiology of IK based on their clinical features can be misleading; indeed, Gram staining and cultures are the gold standard diagnostic tests in these cases [3,4]. It usually takes from 48 h up to weeks for identification and susceptibility testing of bacterial and fungal pathogens (time-to-result period) [5,6]; therefore, during this time frame, an empirical antimicrobial therapy selected upon the ophthalmologist’s clinical experience is generally administered [7].

Most of the times an empirical treatment shows to be a valid support in patients with IK [8]. However, in case of failure, the ophthalmologist will face many diagnostic and therapeutic challenges. At this point, patients might present with an advanced keratitis with blurred clinical features and possibly a concomitant toxic keratitis/medicamentosa [9], leading to the need of a wash out period to reconsider the diagnosis [2] and likely poor clinical outcomes. Moreover, the empirical use of antimicrobial agents might contribute to the rise of multidrug resistances. This has been defined as a global healthcare emergency and has been shown to also pertain to ocular pathogens [10]. Indeed, the need for stricter protocols, an overall more judicious use of antimicrobials and new therapeutic strategies in the field of ophthalmology has already been expressed [10].

Antiseptic agents could be helpful to avoid the use of potentially inappropriate antimicrobial agents while waiting for the microbial testing results. Polyvinylpyrrolidone-iodine (PVP-I) is a disinfectant and antiseptic agent with a broad-spectrum microbiological activity. At concentrations between 5% and 10%, it is the elective infection-prophylactic agent in ocular surgery [11]. Nonetheless, new formulations at lower concentrations (i.e., 0.05–1.5%) have been shown to be even more effective in the treatment of ocular surface infections [12,13,14,15,16].

This seems to be related to the higher availability of the active component of PVP-I itself, which is the diatomic free iodine.

In this study, we assessed the safety and the effectiveness of a nanoemulsion solution containing 0.66% PVP-I administered during the time-to-result period in patients clinically diagnosed with IK.

## 2. Methods

This prospective explorative study was conducted according to the tenets of the Declaration of Helsinki after approval of the local Ethics Committee, and informed consent was obtained from all subjects.

We enrolled patients with signs of IK (i.e., corneal infiltration, corneal epithelial defect and concomitant signs of inflammation) [17] and no ongoing antimicrobial therapy (i.e., antibiotics and/or antimycotics) who accessed the emergency department of the ophthalmic unit at the University Hospital of Verona between February 2021 and July 2021. Only adult patients (18 years of age or more) who agreed to undergo daily follow-up visits and warranted compliance to treatment were enrolled. We excluded patients with impending corneal perforations, endophthalmitis, thyroid diseases, immunosuppressive diseases, PVP-I intolerance, females of childbearing potential/pregnant/breastfeeding and those with protozoan cysts or fungal hyphae detected on in vivo confocal microscopy.

On the day of enrollment (T0), all patients underwent a thorough ophthalmic evaluation and specimen collection for microbiological investigations via corneal swab (CS-Copan eSwabTM, Murrieta, CA, USA) [18,19]. All CSs were performed by the same ophthalmologist (EB) without the use of topical anesthesia [20,21]. A sterile technique was used taking care to avoid any contact with any tissues other than the corneal infiltrate itself. The collected specimens were immediately delivered (Liquid Amies transporting medium) to the microbiological laboratory (Microbiology and Virology Section, Department of Diagnostic and Public Health, University of Verona, Verona, Italy) for culture (Blood Agar, Chocolate Agar and Potato Dextrose Agar) and antimicrobial susceptibility analysis.

During the time-to-result period, patients were monitored daily, and a 0.66% nanoemulsion of PVP-I (Iodim, Medivis, Catania, Italy) was administered: one drop six times/day.

In case of new onset of hypopyon, development of impending corneal perforation or rapidly progressive infiltrate worsening (i.e., deepening or expansion) during the time-to-result period, the patients would have been withdrawn and an empirical therapy or surgical management would have been started.

Clinical findings were evaluated and collected at the slit-lamp (SL). Particularly, we took into account any variation in the corneal ulcer area (mm^2^), infiltrate area (mm^2^) and the ulcer depth (%) occurring during the time-to-result period [22].

We used the edge between the fluorescein-stained and the non-stained cornea to define the ulcer area on cobalt blue light SL photographs, whereas the margin of stromal infiltration was used to mark the infiltrate area on white light SL photographs, taking into account possible prior or concomitant corneal scarring.

All images were analyzed by the same operator (CP) using the Phoenix software (V. 2.6.4.40). The different areas were calculated in square pixels and then converted to square millimeters (mm^2^) following the method introduced by Kriegel et al. [23].

An IK was considered to be completely regressed when the area of both the ulcer and the infiltrate were 0 mm^2^.

Finally, white light SL images at high magnification and anterior-segment optical coherence tomography, high resolution cornea and pachymetry maps (Visante, Carl Zeiss Meditec, DE, V.3.0.1.8) were used to evaluate and to classify the ulcer depth into three categories based on the level of stromal involvement at the thinnest corneal site: 0–33%, 33–67% and >67% [24].

Once the cultural results were available (T1), patients were switched to the specific antimicrobial agent showing the lowest minimal inhibitory concentration (MIC), and the topical 0.66% PVP-I was discontinued. We checked for cultural results every day starting from 48 h after enrollment. All patients went on to be followed up until complete IK resolution was achieved (T last—TL).

Results of descriptive analyses were expressed as means and standard deviation (SD) or median and interquartile range (IQR) for quantitative variables, and as a count and percentages for categorical variables. Evaluations were carried out after grouping the cultural results into four different categories, i.e., “no growth”, “gram-positive”, “gram-negative” and “fungi”. This was necessary because of the low frequencies of each species and for a better interpretation of the results, since each of these large categories has a different pathogenicity.

To assess the safety and the effectiveness of topical PVP-I, we evaluated the difference in both the ulcer and the infiltrate areas and the difference in the ulcer depth between T0 and T1, separately for each category of culture results. This management was considered safe in case of stability or non-statistically significant increase in the above-mentioned parameters, whereas it was considered effective in case of a statistically significant reduction in both the ulcer and the infiltrate area.

Statistical differences were evaluated via the Wilcoxon signed-rank test (R statistical package, version 3.5.1) to compare both ulcer and infiltrate areas. The McNemar–Bowker test was used to compare the depths of the IK (STATA 13.0 statistical package—Stata Corp. LP, College Station, TX, USA). A *p*-value ≤ 0.05 was considered statistically significant.

## 3. Results

We evaluated 25 eyes of 24 patients (10 females and 14 males) with a mean age (SD; range) of 59.5 (±16.71; 30–90) years. A total of 19 out of 25 eyes had clinical history suggestive of IK. Nine were contact lens users, six had previous corneal traumas and four patients had a previous corneal transplant. No patients withdrew from the study between T0 and TL, nor showed any adverse events throughout the study period.

At T0, the mean ulcer and infiltrate areas [median (IQR), mm^2^; range] were 5.7 (5.9; 0.17–18.61) and 3.3 (4.8; 0.17–19.08), respectively, whereas at T1, these became 3.9 (6.7; 0.00–23.18) and 3.1 (2.7; 0.00–13.79), respectively.

The culture was positive in 22 eyes (88.0%): 13 (59.1%) showed Gram-positive bacteria (*n* = 7 *Streptococci* spp, and *n* = 6 *Staphylococci* spp), 5 (22.7%) showed Gram-negative bacteria (all *Pseudomonas Aeruginosa*) and 4 (18.2%) were positive for fungi (all *Candida albicans*). No coinfections were found.

The differences in ulcer and infiltrate areas between T0 and T1 among the different microbiological categories are reported in Table 1.

The mean (SD) time-to-results period was 2 (2.3) and 6 (2.0) days for bacterial and fungal IK, respectively.

The evolution of ulcer depth from T0 to T1 is reported in Table 2.

At T1, nine (36.0%) eyes showed complete resolution of the IK, none of which needed to initiate a specific therapy. Five (55.6%) of these eyes were positive for Gram-positive bacteria, one (11.1%) had a fungal infection and three (33.3%) were in the no growth category.

At T1, Gram-positive IK showed a significant improvement in both the ulcer and the infiltrate areas and a reduction in the ulcer depths.

The mean (SD) healing time (T1-TL, days) was 4 (2.0) for Gram-positive bacteria, 9 (4.1) for Gram-negative bacteria and 9 (5.7) for fungi.

According to the MIC, three eyes with a Gram-positive bacterial infection were treated with topical Vancomycin, two with topical Chloramphenicol, two with topical Netilmicin, one with topical Ciprofloxacin and five eyes did not require any topic antibiotic. Four eyes with a Gram-negative bacterial infection received topical Ceftazidime, and one was treated with Ciprofloxacin. Three out of four fungal infections were treated with Amphotericin B.

## 4. Discussion

The topical 0.66% PVP-I showed to be well tolerated, and it seemed to be effective especially in IKs of Gram-positive bacterial origin. At T1, eyes in this category showed significant improvement in both the ulcer and infiltrate areas and a reduction in ulcer depth. Actually, 38.5% of Gram-positive IK showed complete resolution and did not need to start any antibiotic therapy. On the other hand, while fungal IK showed little improvement in their infiltrate areas and ulcer depths, Gram-negative bacterial IK showed a slight increase in ulcer and infiltrate areas and ulcer depths.

In this regard, it should be emphasized that all the eyes included in the fungal category and all of those with a Gram-negative infection were respectively infected by *Candida albicans* and *Pseudomonas aeruginosa*, two microorganisms with different pathogenetic mechanisms [5,25,26]. *Pseudomonas aeruginosa* is a very invasive bacterium; nevertheless, no statistically significant clinical worsening was found at T1. It should be related to the different time-to-result period required between fungi and bacteria: 48 h for Gram-negative bacteria and 6 days for fungal infections.

Notably, the administration of PVP-I showed effectiveness in 36.0% of cases, 55.6% of which were in the Gram-positive and 11.1% in the fungal category. In the latter group, the empirical antibiotic could have actually worsened the patients’ condition due to the interference with the normal microbial competition [27].

The apparent effectiveness of PVP-I on Gram-positive bacteria is encouraging. Despite being less virulent than Gram-negative bacteria, IK related to these microorganisms are one of the most common causes of ocular surface infections.

A recent randomized controlled trial demonstrated that 1.25% PVP-I eye drops were as effective as topical antibiotics against both gram-negative and gram-positive bacteria [15]. It is conceivable that this concentration of PVP-I has greater efficacy against the *Pseudomonas aeruginosa* than the 0.66% one. However, in this study we utilized the 0.66% concentration because it is the only ophthalmic formulation available on the Italian market.

At T1, three cultural tests displayed no growth, likely due to a low microbial load or to the presence of a sterile infiltrate. In such cases, the topical administration of 0.66% PVP-I represents a further reason to avoid an empirical therapy, which would have been unneeded and potentially detrimental.

It is conceivable that the administration of PVP-I during the time-to-result period might have actually contributed to the relatively fast healing time (T1-TL) displayed in our study [10]. Indeed, while waiting for the cultural results to start the targeted therapy, thanks to its broad-spectrum activity, this antiseptic agent held back the IK of all origins.

Despite the recent increased interest in low dosage Povidone-Iodine as a broad spectrum, low-cost, resistant-free antimicrobic agent [28], there is still limited strong evidence of its in vivo effectiveness in cases of IK [14,15,29,30,31], however, it suggests positive clinical outcomes. Herein, we present the first study to evaluate the role of 0.66% PVP-I during the time-to-results period of IK.

There are several limitations to this study. First, since this was an exploratory study, we included a limited number of patients useful for a preliminary test of our hypothesis. Second, we excluded patients with protozoan cysts or fungal hyphae detected on in vivo confocal microscopy evaluation, and IK showing impending perforations. Therefore, our results cannot be applied to all types of IK.

Further investigations including a larger number of patients in each of the four microbial categories will reduce the variability of the data we found in the present study.

Nonetheless, the administration of topical PVP-I showed to be a safe strategy in patients with IK in order to avoid progression of the infection and is possibly effective against Gram-positive IK.

## Figures and Tables

**Table 1:** Median (IQR) results of both ulcer and infiltrate areas at T0 and T1 for each category after microbial testing results.

	No Growth *n* = 3		
	T0	T1	*p*
Ulcer area (mm^2^)	1.41 (4.49)	0 (0)	0.98
Infiltrate area (mm^2^)	1.40 (2.13)	0 (0)	0.99
	Gram-positive Bacteria *n* = 13		
	T0	T1	*p*
Ulcer area (mm^2^)	4.47 (11.71)	1.33 (6.56)	0.027
Infiltrate area (mm^2^)	1.71 (4.03)	1.10 (4.1)	0.019
	Gram-negative Bacteria *n* = 5		
	T0	T1	*p*
Ulcer area (mm^2^)	3.41 (5.60)	6.35 (9.1)	0.58
Infiltrate area (mm^2^)	3.46 (4.40)	4.18 (1.96)	0.27
	Fungi *n* = 4		
	T0	T1	*p*
Ulcer area (mm^2^)	6.20 (3.90)	6.93 (4.11)	0.98
Infiltrate area (mm^2^)	1.82 (4.14)	1.71 (2.38)	0.98

**Table 2:** Distribution of ulcer depth between T0 and T1, evaluated at the thinnest corneal site.

	T0				T1				*p*
Ulcer Depth	0%	0.33%	0.66%	100%	0%	0.33%	0.66%	100%	
No growth	0	2	1	0	3	0	0	0	0.05
Gram-positive	0	3	5	5	5	5	3	0	0.25
Gram-negative	0	1	3	1	0	0	4	1	1.0
Fungi	0	0	4	0	1	2	2	0	1.0

## Data Availability

The data supporting the conclusions of this article will be made available by the authors, without undue reservation.
